# Socioeconomic disparities in suicide: Causation or confounding?

**DOI:** 10.1371/journal.pone.0243895

**Published:** 2021-01-04

**Authors:** Vincent Lorant, Dharmi Kapadia, Julian Perelman, the DEMETRIQ study group

**Affiliations:** 1 Institute of Health and Society, UCLouvain, Brussels, Belgium; 2 Cathie Marsh Institute for Social Research, University of Manchester, Manchester, United Kingdom; 3 NOVA National School of Public Health, Public Health Research Centre, Universidade NOVA de Lisboa, Lisboa, Portugal; Erasmus Medical Center, NETHERLANDS

## Abstract

**Background:**

Despite an overall reduction in suicide, educational disparities in suicide have not decreased over the last decade. The mechanisms behind educational disparities in suicide, however, remain unclear: low educational status may increase the risk of suicide (“causation”) or low educational status and suicide may share confounders. This paper assesses whether educational disparities in suicide (EDS) are more likely to be due to causation.

**Method:**

The DEMETRIQ study collected and harmonized register-based data on mortality follow-up from forty population censuses from twelve European populations. More than 102,000 suicides were registered over 392 million person-years. Three analyses were carried out. First, we applied an instrumental variable approach that exploits changes in the legislation on compulsory educational age to instrument educational status. Second, we analyzed EDS by age under the hypothesis that increasing EDS over the life cycle supports causation. Finally, we compared EDS in men and women under the assumption that greater EDS in women would support causation.

**Findings:**

The instrumental variable analysis showed no evidence for causation between higher education and suicide, for men or women. The life-cycle analysis showed that the decrease of educational inequalities in suicide between the baseline 1991 period and the 2001 follow-up period was more pronounced and statistically significant in the first three younger age groups. The gender analysis indicated that EDS were systematic and greater in men than in women: the rate ratio of suicide for men with low level of education (RR = 2.51; 95%CI:2.44–2.58) was higher than the rate ratio in women (RR = 1.32; 95CI%:1.26–1.38).

**Interpretation:**

Overall, there was little support for the causation hypothesis, suggesting that the association between education and suicide is confounded. Educational inequalities in suicide should be addressed in early life by early targeting of groups who struggle to complete their education and display higher risk of mental disorder or of mental health vulnerabilities.

## Introduction

Worldwide, suicide accounts for 1.5% of all deaths and ranks as the fourth leading cause of death in high-income countries [1]. Suicide is also socially patterned: it is more frequent in those who are not married [2], in the unemployed [3], and in lower socioeconomic groups [4]. The difference in suicide rates between socioeconomic groups (hereafter, socioeconomic disparities in suicide) is an important topic of research for two reasons. Firstly, these disparities raise questions about the responsiveness of mental health care systems to the needs of the most vulnerable groups. Secondly, despite an overall reduction in suicide, these disparities have not decreased over the last decade either in North America [5,6] or in Europe [7,8].

Socioeconomic disparities in suicide have been investigated in Asia [9–14], North America [5,6,15], Europe [8,16–19], and Australia and New Zealand [20–22]. In addition, two meta-analyses [14,23], one cross-comparative study of fourteen European countries [7], and one narrative review [4] have helped to take stock. In all but one of those studies, people in lower socioeconomic groups were found to be more likely to die by suicide than those in higher socioeconomic groups. Disparities were greatest for occupation-based socioeconomic status (SES), followed by education and, finally, income. The association between low socioeconomic status and suicide is generally more pronounced in men than in women. A Danish case-control study found that suicide risk increased as income decreased (but the same was not true of wealth) and this relationship disappeared once psychiatric history was factored in [18]. Hence, there is a need to see how the risk of suicide is related to low socioeconomic status, controlling for key confounding factors.

Among adults, suicide is an ultimate event and posterior to educational status. Formal education is completed early on in the life cycle and remains time-invariant thereafter. Accordingly, educational disparities in suicide are seen as indicative of a low level of education causing suicide either directly or indirectly through the well-known connection between socioeconomic status and psychiatric disorder [24]. But this overlooks the omitted-variable bias: factors that affect both the risk of low educational attainment and the risk of psychiatric disorder. Suicide is associated with a complex set of factors, which have been reviewed by others [25–27]. It certainly cannot be reduced to psychiatric disorder [28]. Some of these factors may affect both suicide risk factors and educational attainment in the general population: early psychiatric disorder [29,30], impulsiveness [31], childhood behavior [32], childhood adversity [33], and a low level of intelligence [34] are factors that decrease the chance of higher educational achievement and increase the risk of later psychiatric disorder and, thus, of suicide (see S1 Table for a detailed description).

Overall, the direct influence of low educational level on suicide remains a matter of controversy. To take on the challenge of assessing this topic, more insight is needed into the relationship between education and suicide. This paper attempts to address this question, using a theoretically informed approach. We designed three tests aimed at shedding light on the causal relationship between educational level and suicide. First, causation is investigated with a quasi-experimental approach, assessing how changes at the country-level of the legal upper age of compulsory education predict suicide mortality. Second, we analysed how educational inequalities in suicide evolve over age as causation predicts diverging health trajectories between educational groups over the life course. Third, under causation, we expect a steeper gradient for women than for men because women generally have a lower educational level than men, are more exposed to economic hardship and poverty and benefit more from education than men [35–38]. We thus combined theory and empirical analysis of longitudinal data from twelve different populations and we applied these three tests to investigate the association between education and suicide. Together, these analyses throw light on the question of whether educational disparities in suicide are causal.

In seeking an answer to this question, it is important to deliver on the need for an equity lens in suicide prevention [4]. Indeed, if these inequalities result from omitted-variable bias, then early intervention in relation to psychiatric history/vulnerability would make more sense. On the other hand, if a low level of education is a direct determinant of suicide then structural policies aimed at increasing educational opportunities throughout the life course would be needed.

## Materials and methods

### Design

The research question precluded an experimental method and so we looked into observational data for effective signatures of the two hypotheses mentioned above: do the data plead for causation or for confounding? We first used a quasi-experimental approach and performed two additional robustness analyses in order to investigate the causal link between education and suicide. These were then implemented with the longitudinal DEMETRIQ data.

### Data source

The data came from the DEMETRIQ (“Developing Methodologies for Reducing Inequalities in the Determinants of Health”) database, which has been fully described elsewhere [7]. The denominator is composed of population censuses between 1990 and 2007 and linked at the individual level to mortality registers for an average of four years (see Table 1). For Spain-Barcelona, Hungary, Poland, and Estonia the linkage was performed at the group level. For England and Wales, a 1% random sample (the Sample of Anonymized Records) of the population was included. Overall, more than 95% of deaths were successfully linked. The dataset included 12 populations from Northern, Southern, Eastern, and Western Europe: Austria, Belgium, Denmark, England and Wales, Estonia, Finland, Hungary, Italy (Turin Region), Norway, Poland, Spain (Madrid, Barcelona, and Basque regions), and Switzerland (Table 1). For Spain the data from three regions, Barcelona, the Basque region, and Madrid were merged. Italy includes only the region of Turin. The individuals were classified by sex and in 5-year age groups from 35 to 79. Education was harmonized across countries and classified into three groups: low level of education (International Standard Classification for Education–ISCED, 0 to 2, up to lower secondary), medium level of education (ISCED 3–4, upper secondary), and high level of education (ISCED 5+, tertiary education). Deaths were coded using ICD8, 9, or 10. Suicides were identified with the codes for ICD-8 (E950-E959), ICD-9 (E950-E959), and ICD-10 (X60-X84, Y87.0). The deaths included more than 100,000 suicides among 392 million person-years. For all population analyses, number of person-years and number of suicides were weighted so that each population had the same weight.

**Table 1:** Number of person-years and number of suicides per population and census, DEMETRIQ study from twelve European populations, 1991–2001.

Population	Census	Follow-up time (y)	Number of person-years	No. of suicides
Austria	1991	1	3,696,932	1,043
	2001	1	4,248,221	994
Belgium	1991	5	21,308,219	6,030
	2004	1	10,593,107	2,720
Denmark	1991	4	12,194,456	3,910
	1996	4	12,607,023	2,710
	2001	4	13,247,779	2,361
England and Wales	1991	5	1,211,333	95
	1996	5	1,181,362	78
	2001	5	1,280,994	91
	2006	3	938,240	65
Estonia	1987	4	3,609,145	1,379
	1998	4	3,435,255	1,471
Finland	1990	5	12,729,941	4,824
	1995	5	12,418,614	3,948
	2000	5	13,999,113	3,870
	2005	5	13,447,352	3,340
Hungary	1988	3	20,576,688	12,531
	1999	3	21,031,348	9,374
Italy-Turin	1991	5	2,518,551	323
	1996	5	2,217,765	233
	2001	5	2,460,183	234
	2006	4	1,822,737	154
Norway	1990	5	8,033,047	1,470
	1995	6	8,181,245	1,251
	2001	5	8,956,862	1,277
	2006	3	4,889,983	688
Poland	1991	2	41,567,370	9,871
	2001	2	42,980,313	10,478
Spain (3 regions)	1992	4	4,290,318	368
	1996	3	9,230,787	605
	1997	4	4,146,288	365
	2001	4	10,292,666	693
	2002	4	4,347,257	399
	2007	3	3,537,654	358
Switzerland	1990	5	13,714,409	4,130
	1995	5	13,264,927	3,684
	2000	5	14,208,708	3,423
	2005	3	8,306,439	1,925
Total			392,722,631	102,763

### Data analysis

Two-stage regression (2SLS) is a quasi-experimental approach, which we used to identify predictors of educational status that are unlikely to be associated with confounders. Here, we first looked for instrumental variables and found that changes in the legal upper age of compulsory education could be an instrument of individual educational status, an approach that had been used by others [39–42]. We surveyed educational reforms in the DEMETRIQ countries and found that twelve countries had carried out educational reforms: one of these (Belgium) occurred too early to be captured in our dataset (see supplementary tables) and one (Finland) occurred too late. We then checked whether these reforms could be a good instrument: using graphs, we identified discontinuity in the distribution of those with a high level of education as a response to the reforms and we excluded countries where the reform was not associated with a statistically significant increase in the percentage of individuals with medium or high levels of education after the reform (Austria, Denmark, and Finland). The S2 Table describes the educational reform per country, together with the sources of information and the effect of the reform on the percentage of individuals with a medium level of education. S1 Fig displays the change in the educational level following the pivotal year of the education reform. We were left with eight countries in which 54.4% of person-years were exposed to the reform and 45.6% were not. We ran probit models of high education level predicted by country, sex, age, and dummies indicating when the age of compulsory education was increased. The predicted value of a high educational level was then computed and was retained for the second-stage analysis. Endogeneity of high educational level was also tested with the Durbin-Wu-Hausman (DWH) test. Two-stage least square regressions were performed with suicide mortality rate as the dependent variable and the predicted educational level as an instrumental variable for individual education, in addition to other covariates. Separate analyses were run for men and women.

In addition, two complementary analyses were performed according to age and to gender. The causation theory of cumulative disadvantage predicts diverging health trajectories between educational groups over a lifetime [43,44]. Under cumulative disadvantage, disparities in suicide would increase because educational status brings benefit at each stage of the life cycle, thus leading to increasing differences between the different educational groups. Alternatively, health trajectories between educational groups will converge in later life cycles: this is because, at each stage of the life cycle, suicide removes a vulnerable subgroup among those with less education, rendering more similar the composition of the different educational groups regarding these confounding factors.

We thus modeled the number of suicides per person-year with a Poisson regression using educational group, age, and the interaction of education and age, controlling for the census period, sex, and country as dummies. Having a college degree, however, means different things in younger age groups than in older age groups. To control for this cohort effect, we included the age-specific relative rank of each educational group as an additional control.

Comparing suicide inequalities across different age groups does not, however, disentangle cohort from age effects. As we were able to include at least with at least two repeated follow-ups per age group for several populations, we were able to construct a pseudo-panel at the group level as proposed by Deaton and others [45,46]. We constructed baseline groups according to education (low, middle, high), gender, birth cohort of five years at the first period of reference (1990/91). Each baseline group was then matched with the second period, by aging each baseline group according to the time span between the first census and the second census (10 years). Because we counted with age group of 5 years, we retained only countries for which we had at least two censuses 10 years apart. For this analysis, we retained Denmark, Finland, and Norway because the data covered the whole country, for a longer period, and had better suicide coding. We ran a Poisson regression, and rate ratios were computed for each educational group, age, and period (baseline census and follow-up census), controlling for country, gender, and age at baseline. Pooled analyses were weighted so that each population-period had the same weight.

**Fig 1:**
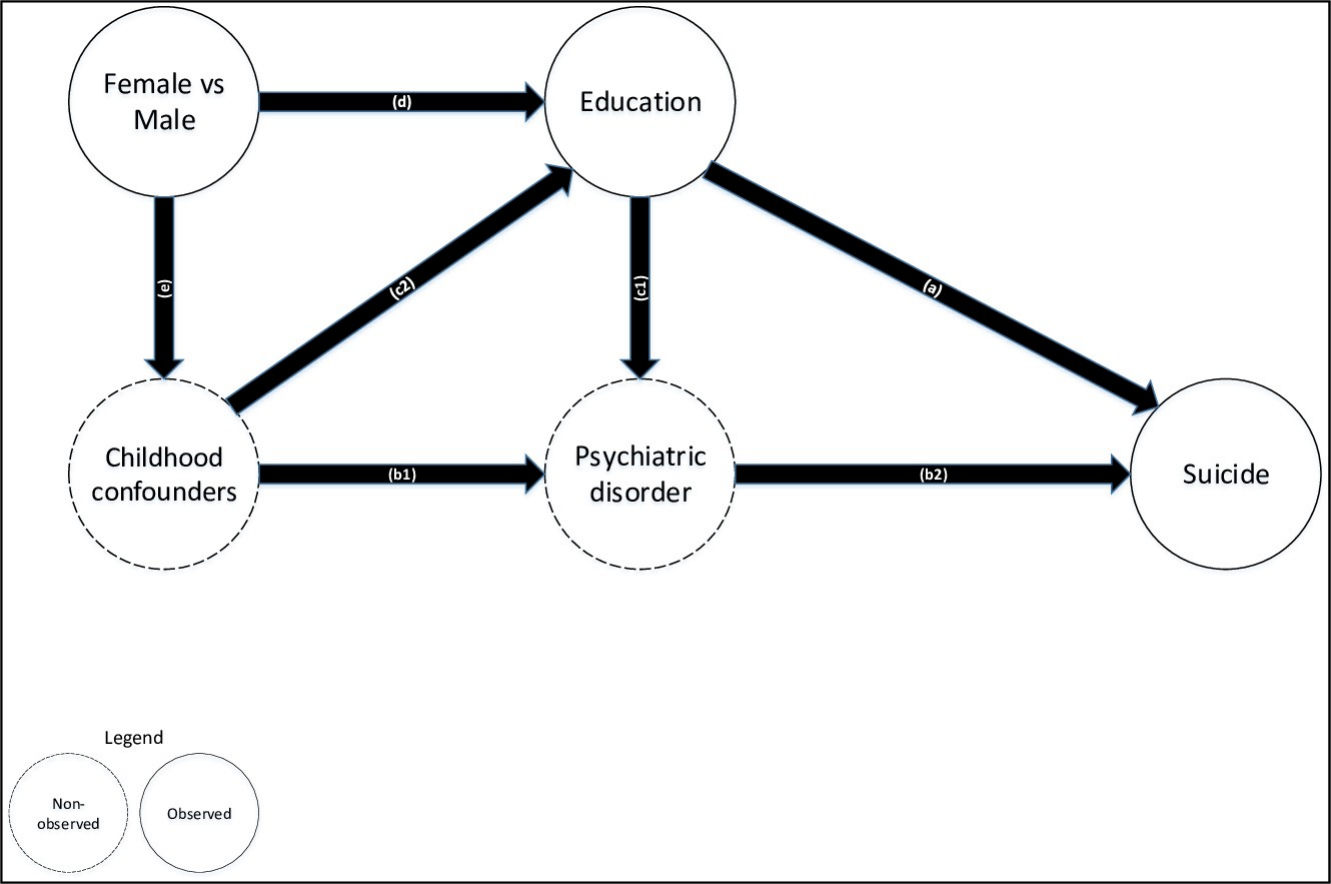
Theoretical model of educational disparities in suicide, with unobserved and observed variables in the DEMETRIQ dataset.

Our third analysis relied on the gendered difference in socioeconomic disparities in suicide. As explained above, the relationship between socioeconomic status, suicide, and the confounders differs both in terms of sign and magnitude for men and women (see Fig 1). Women have generally had a lower educational level than men and they are more exposed to economic hardship and poverty [35–37]. In addition, the economic gain from education is higher for women than for men [38]. Accordingly, the causal relationship between education and suicide (a and c1*b2, Fig 1) is expected to be more negative for women than for men. As far as the confounding factors are concerned, psychiatric disorders in childhood are more prevalent in boys than in girls [29,30]; impulsiveness, a key trigger of suicide, is more frequent in men; there are no clear gender differences in childhood adversity, with the exception of sexual abuse, which is more frequently experienced by girls [47–49]. Early psychiatric disorder in childhood or adolescence has been investigated across sex groups: Costello 2003 and Merikangas 2010 found that boys were more likely to have a psychiatric disorder than girls. Boys and girls differ in the type of disorder: conduct or behavioral disorder are more frequent in boys, whereas depression is more frequent in girls [29,30]. Hence, these confounding factors may be considered slightly more frequent in boys than in girls: this implies that the arrow (e, in Fig 1) is either negative or close to zero. As a conclusion, we expect women, as compared to men, to be more vulnerable to causation (because of d < 0 and because c1 is higher in absolute value for women than for men) but less vulnerable to confounding effects (e is either < 0 or →0). From a statistical point of view, our analysis rests on testing the interaction of educational status and gender in relation to the number of suicides per person-year using Poisson regressions, for each country and for the pooled dataset (weighted analysis with countries as dummies).

Each hypothesis is associated with different expectations: causation is supported by a negative effect of instrumented education on suicide in the 2SLS. Causation predicts a diverging trend in educational disparities in suicide; causation leads to a steeper gradient for women than for men. Together, these analyses throw light on the question of whether educational disparities in suicide are more likely to be causal or not.

All analyses were carried out with STATA 15.1 and SAS 9.4.

## Results

### Quasi-experimental approach

Results of the 2SLS are displayed in Table 2 for the first stage (upper panel) and for the second stage (lower panel). A high level of education was more likely when the legal upper age limit for compulsory education was increased; it was more frequent in some countries (i.e. Switzerland and Norway) than others; it was higher at a younger age. Interestingly, the change in the upper age limit for compulsory education was associated with a greater increase in the proportion with a high level of education among women (Beta = 0.244) than among men (Beta = 0.124). The McFadden pseudo-R2 were 0.16 for men and 0.14 for women; the robust test of weak instrument was rejected (F(2,9) = 12.7, *p* = 0.0029) for both gender groups (Men: 10.23, *p*<0.01; Women: 11.12, *p*<0.01). Exogeneity of education was rejected with a DWH chi-square of endogeneity of 11.88 (*P* ≤ 0.001) for men and 55.63 (*P* ≤ 0.001) for women.

**Table 2:** Two-stage least square estimates, DEMETRIQ study of twelve European populations, 1991–2001.

	Both genders	Men	Women
	Coefficient (SE)	Coefficient (SE)	Coefficient (SE)
Covariate			
Predictor of medium or high level of education: first-stage probit estimate			
Change in upper age limit for compulsory education (dummy)	0.181***	0.123***	0.244***
	(0.000236)	(0.000346)	(0.000325)
Women (ref = men)	-0.0974***		
	(0.000150)		
Age (y.)	-0.0223***	-0.0166***	-0.0270***
	(9.21e-06)	(1.37e-05)	(1.26e-05)
Intercept	1.499***	1.487***	1.415***
	(0.000737)	(0.00110)	(0.00101)
F-test of Weak instrument	12.69***	10.23***	11.12***
Predictors of suicide mortality: second-stage estimates			
Medium or high education level	-0.297(-1.896, 1.302)	0.515(-0.982, 2.012)	-0.419(-1.756, 0.918)
	(0.816)	(0.764)	(0.682)
Women (ref = men)	-1.135***		
	(0.139)		
Intercept	-7.880***	-8.503***	-8.856***
	(0.619)	(0.569)	(0.544)
N	272,906,066	129,203,487	143,702,579

¶Robust standard errors in parentheses

*** *P* < 0.01

** *P* < 0.05

* *P* < 0.1.

‡Controlled for 5y age groups and country dummies.

In the second stage (Table 2, lower panel), suicide mortality was not significantly associated with a high level of education, for either men or women. Suicide was lower among women than among men and lower in southern countries than in eastern countries. The same results were found with a medium level of education.

These estimates were performed on countries (Belgium, England and Wales, Estonia, Hungary, Italy, Norway, Poland, Spain, and Switzerland) with a positive effect of the instrument in the first-stage equation. Three countries had a negative effect and were thus excluded: Austria, Denmark, and Finland.

### Educational disparities in suicide by age

**Fig 2:**
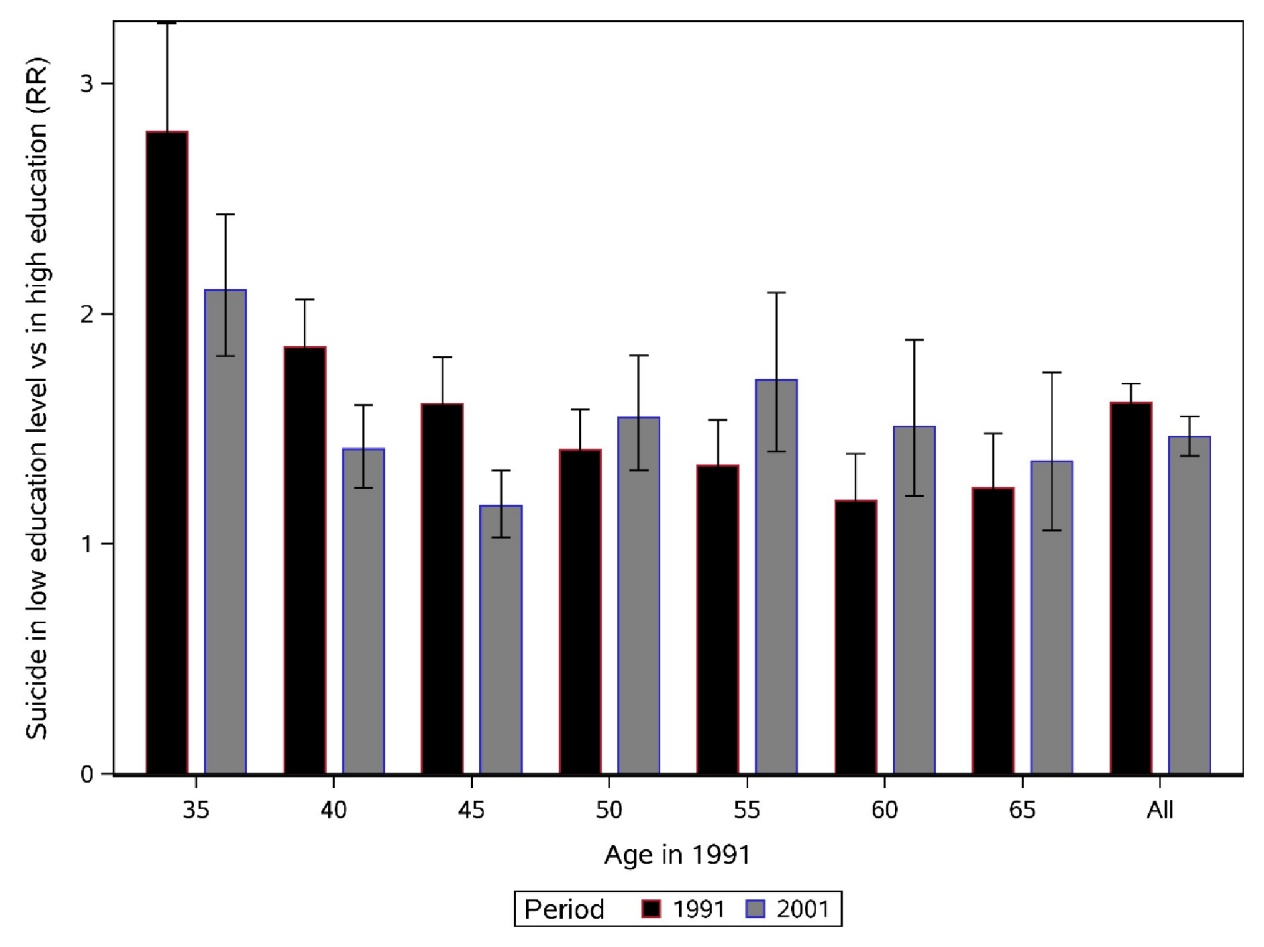
Educational disparities in suicide across age groups and period: Rate ratio of suicide, pooled and weighted Poisson regression analysis of Denmark, Finland, and Norway, 1991–2001.

Across all age groups, the risk of suicide among those with the lowest level of education, compared to the most highly educated, was 1.61 (95%CI:1.53, 1.70) at baseline and decreased to 1.46 (95%CI: 1.38, 1.55) ten years later, a decrease which was statistically significant (Chi = 6.07, *p* = 0.014). Fig 2 displays the pooled rate ratio (RR) of suicide for each age and period: the decrease of educational inequalities in suicide between the baseline 1991 period and the 2001 follow-up period was more pronounced and statistically significant in the first three younger age groups. For example, among those aged 35–39, inequalities decreased from 2.79 to 2.10 between 1991 and 2001.

### Educational disparities in suicide for men and women

**Fig 3:**
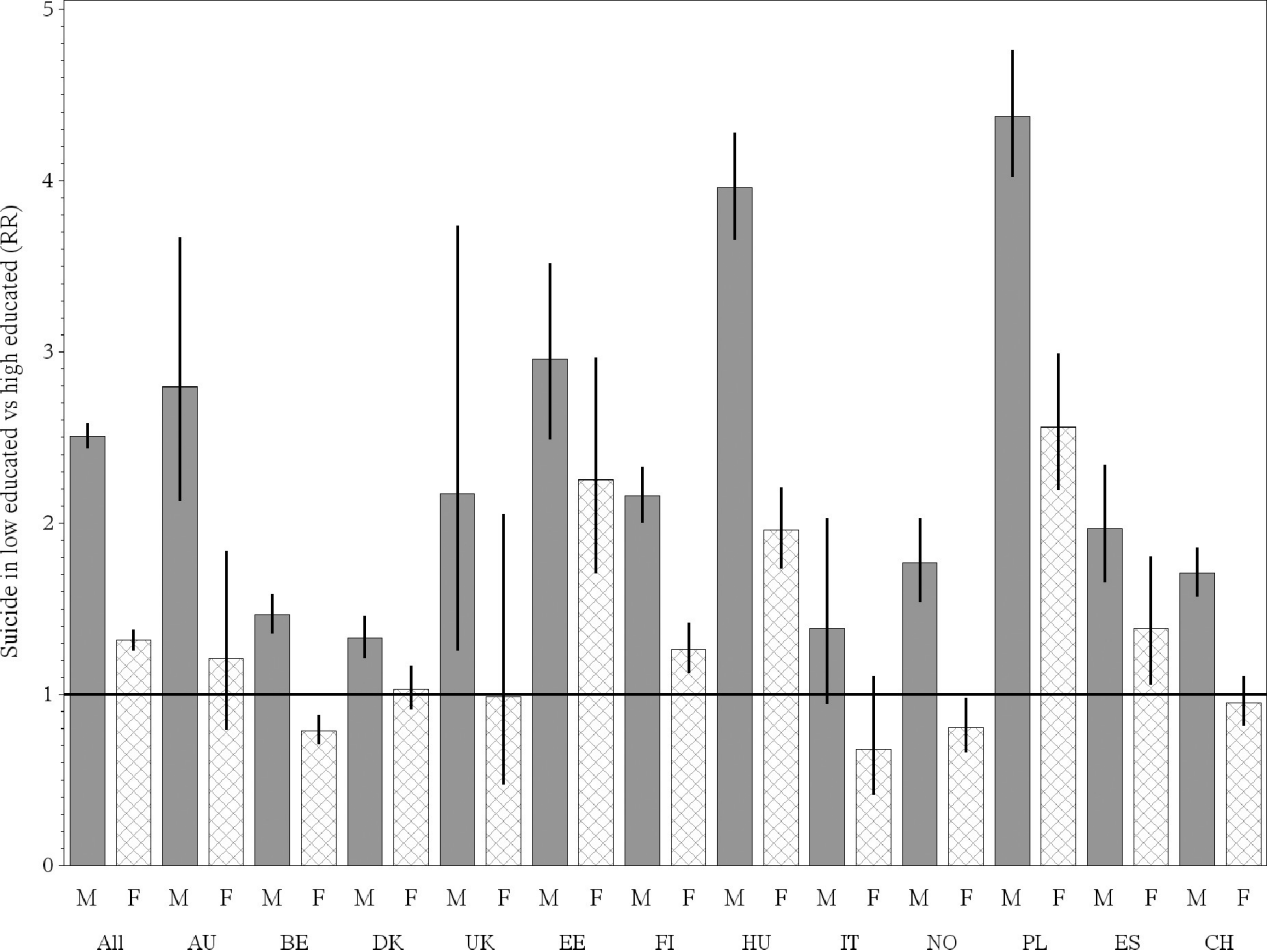
Educational disparities in suicide per population and gender (F = Women, M = Men): Pooled and weighted results and population-specific risk ratio of suicide from the Poisson regressions, DEMETRIQ study from twelve European populations, 1991–2001.

The educational disparities in suicide for men (M) and women (F) are displayed in Fig 3. In the pooled analysis, the risk of suicide for women with a low level of education compared to those with a high level was 1.32 (95CI%:1.26, 1.38); in men, this ratio was higher (2.51; 95%CI:2.44, 2.58). In all countries except two (England-Wales and Estonia), educational disparity was statistically significantly higher in men than in women (see last column of S3 Table).

## Discussion

### Main findings

This paper contributes novel information on the association between education and suicide. The instrumental variable showed that group-level predictors of a high level of education were not associated with suicide. In addition, educational disparities in suicide decreased with age, thus not supporting causation. This general finding should, however, be nuanced, as disparities in suicide were larger between 55 and 65 years. As these disparities were more systematic and marked for men than for women, causation was again less likely. Overall, this paper found little support for a causal relationship between education and suicide.

### Interpretation

Several reviews have looked anew at the social determinants of suicide and at the debate about which models are relevant [4,50,51]. From a psychiatry perspective, two models compete as far as depression is concerned, one in which psychosocial factors are determinants of both depression and suicide, the other in which depression affects both psychosocial factors and suicide. In their review of current empirical evidence related to suicide prevention, Hegerl and Heinz [52] found more support for the latter than for the former, lending support to our conclusions.

Our results are equally consistent with previous empirical studies. Our instrumental analysis compares well with another study that investigated municipal differences in relation to raising the upper age limit for compulsory education by one year between 1949 and 1962 in Sweden. To the best of our knowledge, that was the only instrumental variable approach to provide cause-specific mortality results, including suicide and intentional self-harm. It found no effect for all-causes mortality and no effect for suicide/intentional self-harm in either men or women [40]. Besides the Danish register study, a recent case-control study from Finland points to the same conclusions. The Finnish study found a reverse gradient, with suicide occurring slightly more frequently among those with a high level of education than among those with a low level, once depression severity and other clinical features were accounted for [52].

### Limitations

The DEMETRIQ dataset lacked information related to the possible confounders. The literature provides some insights, which are summarized in S1 Table, in which we have excluded factors posterior to education (i.e. unemployment and marital status) [3]. Candidates for omitted-variable bias include: (a) childhood or adolescent psychiatric disorder, (b) latent factors such as impulsiveness and intelligence [34], and (c) childhood adversity. All these variables have been found to increase both the risk of suicide and poor educational achievement.

The instrumental variable approach had some limitations: we had categories of education and not completed years of education; thus, our estimate is possibly a lower bound. Finally, for some countries, raising the upper age limit for compulsory education was a poor instrument of education status.

In addition, education is a time-invariant marker of socioeconomic status. It has the advantage of being insensitive to later changes in psychiatric condition but, as a consequence, does not capture the full range of socioeconomic opportunities such as income, which may show a different pattern for the two hypotheses tested here [53]. The underreporting of suicide in registers is another limitation. Although suicide is certainly underreported in some countries, a previous sensitivity analysis showed that the magnitude of educational disparities in suicide was only weakly affected by underreporting when considering countries with more reliable data or when including deaths categorized as “injuries–unknown whether intentional” as covert suicide [54,55].

As far as the life-course analysis was concerned, it could not do justice to the dynamic relationships involved in these disparities [56]. The mechanisms driving the association between education and mental health are different across the life course and also involve inter-generational processes [57,58]. The decreasing educational inequalities in suicide with age might also be the result of the differential role that education plays in different life stages. Education is an important dimension of social stratification in young adults, but may be less relevant in older age where wealth and housing tenure are more important. Also, educational disparities in suicide may be lower in women in part because suicide is much less frequent among women than among men or because the relationship between education and suicide is different in men and women. So there may be a limit to the attempt to separate the gender gradient in suicide from the educational gradient in suicide. Yet, even in countries where educational disparities in suicide were more pronounced (i.e. eastern European countries), disparities for men always exceeded disparities for women. Finally, this study was performed with European populations, where, on the whole, income inequality is rather low and health care coverage is quite extensive compared to other continents.

### Conclusions

The higher risk of suicide in less educated individuals may be due to early-life factors reducing educational opportunities and increasing mental health vulnerabilities. Paying more attention to young men who are struggling to complete their educational track and who have mental health vulnerabilities should become a priority in order to tackle educational inequalities in suicide.

## Supporting information

S1 Fig Percentage of those with a high level of education in the population by number of years before/after the educational reform, per country. (TIF)

S1 Table Factors affecting the risk of suicide and low educational level: A brief overview of the literature. (DOCX)

S2 Table Changes in compulsory schooling legislation. ¶ Effect of exposure to the reform in the following logit equation: medium high level of education = age group + Census year + Sex + exposure to the reform.
(DOCX)

S3 Table Results of Test 2, educational disparities in suicide by gender and population, DEMETRIQ study from twelve European populations, 1991–2001. ¶ All estimates are controlled for sex, 5y. age group, and year of the census. Results for all countries are weighted and controlled by country dummies.
(DOCX)

## References

[1] Mortality GBD, Causes of Death C. Global, regional, and national age-sex specific all-cause and cause-specific mortality for 240 causes of death, 1990–2013: a systematic analysis for the Global Burden of Disease Study 2013. Lancet. 2015;385(9963):117–71. 10.1016/S0140-6736(14)61682-2 25530442 PMC4340604

[2] Lorant V, Kunst AE, Huisman M, Bopp M, Mackenbach J, Group EUW. A European comparative study of marital status and socio-economic inequalities in suicide. Social science & medicine. 2005;60(11):2431–41. 10.1016/j.socscimed.2004.11.033 15814169

[3] Milner A, Page A, Lamontagne AD. Cause and effect in studies on unemployment, mental health and suicide: A meta-analytic and conceptual review. Psychological Medicine. 2014;44(5):909–17. 10.1017/S0033291713001621 23834819

[4] Platt S. Inequalities and Suicidal Behavior. The International Handbook of Suicide Prevention: Second Edition 2016 p. 258–83.

[5] Phillips JA, Hempstead K. Differences in U.S. Suicide Rates by Educational Attainment, 2000–2014. American Journal of Preventive Medicine. 2017;53(4):e123–e30. 10.1016/j.amepre.2017.04.010 28756896

[6] Burrows S, Auger N, Roy M, Alix C. Socio-economic inequalities in suicide attempts and suicide mortality in Québec, Canada, 1990–2005. Public Health. 2010;124(2):78–85. 10.1016/j.puhe.2010.01.008 20181370

[7] Lorant V, de Gelder R, Kapadia D, Borrell C, Kalediene R, Kovacs K, et al Socioeconomic inequalities in suicide in Europe: the widening gap. Br J Psychiatry. 2018;212(6):356–61. 10.1192/bjp.2017.32 29786492

[8] Mäki NE, Martikainen PT. Socioeconomic differences in suicide mortality by sex in Finland in 1971–2000: A register-based study of trends, levels, and life expectancy differences. Scandinavian Journal of Public Health. 2007;35(4):387–95. 10.1080/14034940701219618 17786802

[9] Yoshioka E. Suicide, socio-economic inequalities, gender, and psychiatric disorders: Commentary: Educational levels and risk of suicide in Japan: The Japan Public Health Center Study (JPHC) Cohort I. Journal of Epidemiology. 2016;26(6):277–8. 10.2188/jea.JE20160082 27265746 PMC4884894

[10] Tsai JF. Socioeconomic factors outweigh climate in the regional difference of suicide death rate in Taiwan. Psychiatry Research. 2010;179(2):212–6. 10.1016/j.psychres.2008.06.044 20483166

[11] Fukuda Y, Nakamura K, Takano T. Cause-specific mortality differences across socioeconomic position of municipalities in Japan, 1973–1977 and 1993–1998: Increased importance of injury and suicide in inequality for ages under 75. International Journal of Epidemiology. 2005;34(1):100–9. 10.1093/ije/dyh283 15561754

[12] Veisani Y, Delpisheh A, Sayehmiri K, Moradi G, Hassanzadeh J. Decomposing socioeconomic inequality determinants in suicide deaths in Iran: A concentration index approach. Korean Journal of Family Medicine. 2017;38(3):135–40. 10.4082/kjfm.2017.38.3.135 28572889 PMC5451447

[13] Kim MH, Jung-Choi K, Jun HJ, Kawachi I. Socioeconomic inequalities in suicidal ideation, parasuicides, and completed suicides in South Korea. Social Science and Medicine. 2010;70(8):1254–61. 10.1016/j.socscimed.2010.01.004 20163900

[14] Li Y, Li Y, Cao J. Factors associated with suicidal behaviors in mainland China: A meta-analysis. BMC Public Health. 2012;12(1). 10.1186/1471-2458-12-524 22800121 PMC3490836

[15] Denney JT, Rogers RG, Krueger PM, Wadsworth T. Adult suicide mortality in the United States: Marital status, family size, socioeconomic status, and differences by sex. Social Science Quarterly. 2009;90(5):1167–85. 10.1111/j.1540-6237.2009.00652.x 20161640 PMC2818047

[16] Lorant V, Kunst AE, Huisman M, Costa G, Mackenbach J. Socio-economic inequalities in suicide: A European comparative study. British Journal of Psychiatry. 2005;187(JULY):49–54. 10.1192/bjp.187.1.49 15994571

[17] Middleton N, Whitley E, Frankel S, Dorling D, Sterne J, Gunnell D. Suicide risk in small areas in England and Wales, 1991–1993. Social Psychiatry and Psychiatric Epidemiology. 2004;39(1):45–52. 10.1007/s00127-004-0707-y 15022046

[18] Qin P, Agerbo E, Mortensen PB. Suicide risk in relation to socioeconomic, demographic, psychiatric, and familial factors: a national register-based study of all suicides in Denmark, 1981–1997. The American journal of psychiatry. 2003;160(4):765–72. 10.1176/appi.ajp.160.4.765 12668367

[19] Qin P, Agerbo E, Westergard-Nielsen N, Eriksson T, Mortensen PB. Gender differences in risk factors for suicide in Denmark. Br J Psychiatry. 2000;177:546–50. 10.1192/bjp.177.6.546 11104395

[20] Blakely T, Woodward A, Pearce N, Salmond C, Kiro C, Davis P. Socio-economic factors and mortality among 25–64 year olds followed from 1991 to 1994: the New Zealand Census-Mortality Study. N Z Med J. 2002;115(1149):93–7. 11999230

[21] Taylor R, Page A, Morrell S, Harrison J, Carter G. Mental health and socio-economic variations in Australian suicide. Social Science and Medicine. 2005;61(7):1551–9. 10.1016/j.socscimed.2005.02.009 16005786

[22] Page A, Morrell S, Taylor R, Carter G, Dudley M. Divergent trends in suicide by socio-economic status in Australia. Social Psychiatry and Psychiatric Epidemiology. 2006;41(11):911–7. 10.1007/s00127-006-0112-9 16951920

[23] Li Z, Page A, Martin G, Taylor R. Attributable risk of psychiatric and socio-economic factors for suicide from individual-level, population-based studies: A systematic review. Social Science and Medicine. 2011;72(4):608–16. 10.1016/j.socscimed.2010.11.008 21211874

[24] Lorant V, Deliege D, Eaton W, Robert A, Philippot P, Ansseau M. Socioeconomic inequalities in depression: a meta-analysis. American journal of epidemiology. 2003;157(2):98–112. 10.1093/aje/kwf182 12522017

[25] Nock MK, Borges G, Bromet EJ, Cha CB, Kessler RC, Lee S. Suicide and suicidal behavior. Epidemiologic Reviews. 2008;30(1):133–54. 10.1093/epirev/mxn002 18653727 PMC2576496

[26] Hawton K, van Heeringen K. Suicide. Lancet. 2009;373(9672):1372–81. 10.1016/S0140-6736(09)60372-X 19376453

[27] Mann JJ, Apter A, Bertolote J, Beautrais A, Currier D, Haas A, et al Suicide prevention strategies: A systematic review. Journal of the American Medical Association. 2005;294(16):2064–74. 10.1001/jama.294.16.2064 16249421

[28] Saxena S, Krug EG, Chestnov O, World Health Organization. Department of Mental Health and Substance Abuse. Preventing suicide: a global imperative. Geneva: World Health Organization; 2014. 89 pages p.

[29] Costello EJ, Mustillo S, Erkanli A, Keeler G, Angold A. Prevalence and development of psychiatric disorders in childhood and adolescence. Archives of General Psychiatry. 2003;60(8):837–44. 10.1001/archpsyc.60.8.837 12912767

[30] Merikangas KR, He JP, Brody D, Fisher PW, Bourdon K, Koretz DS. Prevalence and Treatment of Mental Disorders Among US Children in the 2001–2004 NHANES. Pediatrics. 2010;125(1):75–81. 10.1542/peds.2008-2598 20008426 PMC2938794

[31] Cross CP, Copping LT, Campbell A. Sex Differences in Impulsivity: A Meta-Analysis. Psychological Bulletin. 2011;137(1):97–130. 10.1037/a0021591 21219058

[32] Vergunst F, Tremblay RE, Nagin D, Algan Y, Beasley E, Park J, et al Association Between Childhood Behaviors and Adult Employment Earnings in Canada. JAMA Psychiatry. 2019;76(10):1044–51. 10.1001/jamapsychiatry.2019.1326 31215972 PMC6584893

[33] Straatmann VS, Lai E, Lange T, Campbell MC, Wickham S, Andersen A-MN, et al How do early-life factors explain social inequalities in adolescent mental health? Findings from the UK Millennium Cohort Study. 2019;73(11):1049–60. 10.1136/jech-2019-212367 31492761 PMC6877708

[34] Gunnell D, Magnusson PK, Rasmussen F. Low intelligence test scores in 18 year old men and risk of suicide: cohort study. BMJ. 2005;330(7484):167 10.1136/bmj.38310.473565.8F 15615767 PMC544986

[35] OECD. The ABC of Gender Equality in Education: Aptitude, Behaviour, Confidence. Paris: OECD; 2015.

[36] Mandel H, Semyonov M. Family policies, wage structures, and gender gaps: Sources of earnings inequality in 20 countries. American Sociological Review. 2005;70(6):949–67.

[37] OECD. In it together: why less inequality benefits all. Paris: OECD Publishing; 2015.

[38] DiPrete TA, Buchmann C. Gender-specific trends in the value of education and the emerging gender gap in college completion. Demography. 2006;43(1):1–24. 10.1353/dem.2006.0003 16579206

[39] Lleras-Muney A. The relationship between education and adult mortality in the United States. Review of Economic Studies. 2005;72(1):189–221.

[40] Lager ACJ, Torssander J. Causal effect of education on mortality in a quasi-experiment on 1.2 million Swedes. Proceedings of the National Academy of Sciences of the United States of America. 2012;109(22):8461–6. 10.1073/pnas.1105839109 22586112 PMC3365223

[41] Clark D, Royer H. The effect of education on adult mortality and health: Evidence from Britain. American Economic Review. 2013;103(6):2087–120. 10.1257/aer.103.6.2087 29533059

[42] Gathmann C, Jurges H, Reinhold S. Compulsory schooling reforms, education and mortality in twentieth century Europe. Social science & medicine. 2015;127:74–82. 10.1016/j.socscimed.2014.01.037 24560098

[43] Cullati S. Socioeconomic inequalities in health trajectories in Switzerland: are trajectories diverging as people age? Sociology of Health & Illness. 2015;37(5):745–64. 10.1111/1467-9566.12232 25683678

[44] Sacker A, Worts D, McDonough P. Social influences on trajectories of self-rated health: evidence from Britain, Germany, Denmark and the USA. J Epidemiol Community Health. 2011;65(2):130–6. 10.1136/jech.2009.091199 19996360

[45] Deaton AS, Paxson CH. Aging and inequality in income and health. American Economic Review. 1998;88(2):248–53.

[46] Baeten S, Van Ourti T, van Doorslaer E. The socioeconomic health gradient across the life cycle: What role for selective mortality and institutionalization? Social Science and Medicine. 2013;97:66–74. 10.1016/j.socscimed.2013.08.019 24161090 PMC3831059

[47] Stoltenborgh M, Bakermans-Kranenburg MJ, Alink LRA, van Ijzendoorn MH. The Prevalence of Child Maltreatment across the Globe: Review of a Series of Meta-Analyses. Child Abuse Review. 2015;24(1):37–50.

[48] Stoltenborgh M, Bakermans-Kranenburg MJ, Van Ijzendoorn MH. The neglect of child neglect: A meta-analytic review of the prevalence of neglect. Social Psychiatry and Psychiatric Epidemiology. 2013;48(3):345–55. 10.1007/s00127-012-0549-y 22797133 PMC3568479

[49] Bjorkenstam C, Kosidou K, Bjorkenstam E. Childhood adversity and risk of suicide: cohort study of 548 721 adolescents and young adults in Sweden. BMJ. 2017;357:j1334 10.1136/bmj.j1334 28424157

[50] Hegerl U, Heinz I. Reflections on causes of suicidal behaviour. Epidemiol Psychiatr Sci. 2018:1–4. 10.1017/S2045796018000562 30322419 PMC6998919

[51] Wray M, Colen C, Pescosolido B. The sociology of suicide. Annual Review of Sociology 2011 p. 505–28.

[52] Aaltonen KI, Isometsä E, Sund R, Pirkola S. Risk factors for suicide in depression in Finland: first-hospitalized patients followed up to 24 years. Acta Psychiatrica Scandinavica. 2019;139(2):154–63. 10.1111/acps.12990 30480317

[53] Sareen J, Afifi TO, McMillan KA, Asmundson GJG. Relationship between household income and mental disorders: Findings from a population-based longitudinal study. Archives of General Psychiatry. 2011;68(4):419–27. 10.1001/archgenpsychiatry.2011.15 21464366

[54] Reynders A, Scheerder G, Van Audenhove C. The reliability of suicide rates: An analysis of railway suicides from two sources in fifteen European countries. Journal of Affective Disorders. 2011;131(1–3):120–7. 10.1016/j.jad.2010.11.003 21129779

[55] Vaernik P, Sisask M, Vaernik A, Arensman E, Van Audenhove C, van der Feltz-Cornelis CM, et al Validity of suicide statistics in Europe in relation to undetermined deaths: developing the 2–20 benchmark. Injury Prevention. 2012;18(5):321–5. 10.1136/injuryprev-2011-040070 22157205

[56] Chandler A. Socioeconomic inequalities of suicide: Sociological and psychological intersections. European Journal of Social Theory. 2018.

[57] Miech RA, Shanahan MJ. Socioeconomic status and depression over the life course. Journal of Health and social behavior. 2000;41(2):162–76.

[58] Johnson JG, Cohen P, Dohrenwend BP, Link BG, Brook JS. A longitudinal investigation of social causation and social selection processes involved in the association between socioeconomic status and psychiatric disorders. Journal of Abnormal Psychology. 1999;108(3):490–9. 10.1037//0021-843x.108.3.490 10466273

