# Supplementary figures and images for: Socioeconomic disparities in suicide: Causation or confounding?
Source: PLoS One. 2021 Jan 4;16(1):e0243895. doi: 10.1371/journal.pone.0243895 (PMC7781379; doi:10.1371/journal.pone.0243895)

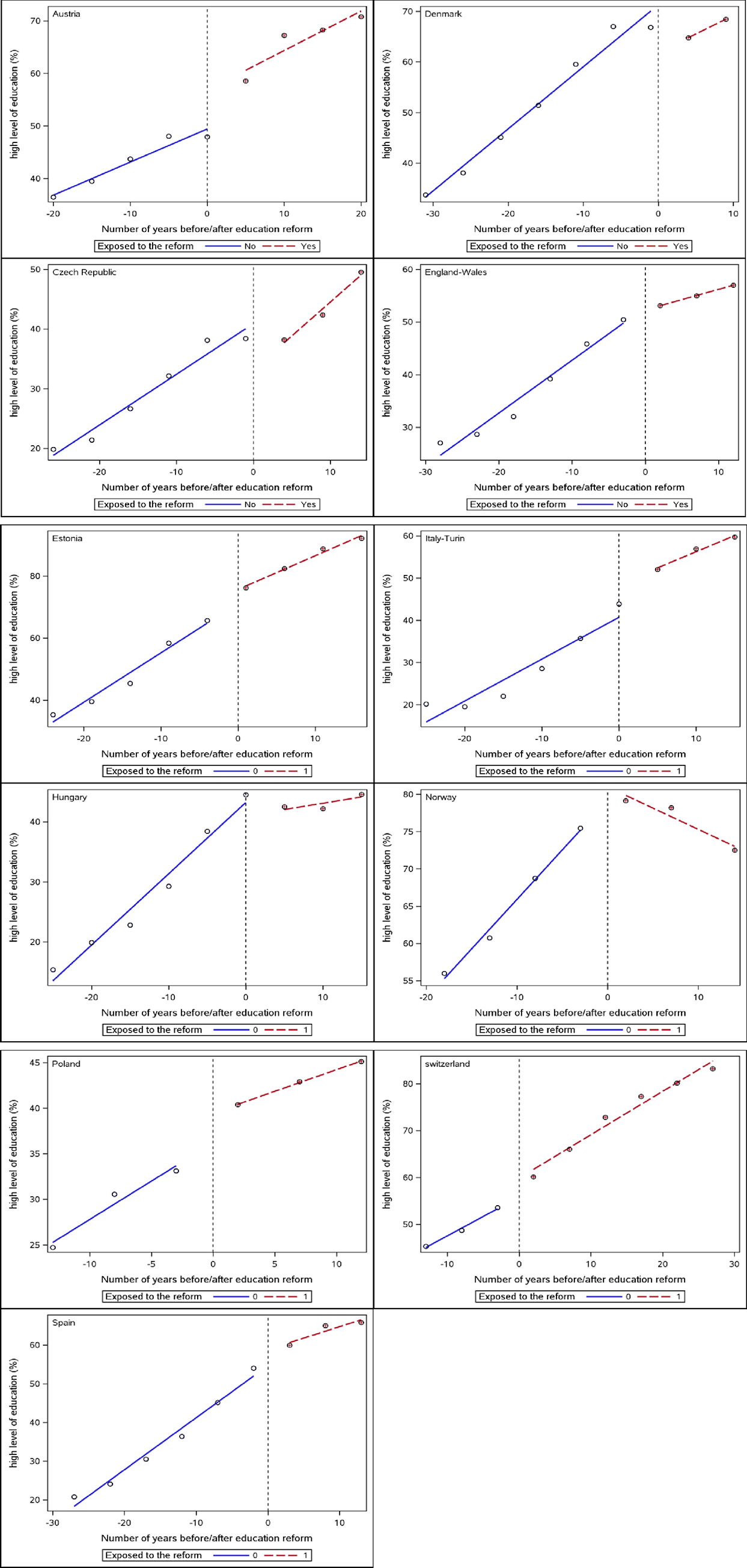

Supplement: S1 Fig — (TIF) [file pone.0243895.s001.tif]
