# Supplementary material for: Socioeconomic disparities in suicide: Causation or confounding?
Source: PLoS One. 2021 Jan 4;16(1):e0243895. doi: 10.1371/journal.pone.0243895 (PMC7781379; doi:10.1371/journal.pone.0243895)
Supplement: S1 Table — (DOCX) [file pone.0243895.s002.docx]

S1 Table. Factors affecting the risk of suicide and low educational level: A brief overview of the literature

| Factor | Factor affecting the risk of suicide | Factor affecting the risk of low level of education | Gender differences |
| --- | --- | --- | --- |
| Psychiatric disorder | Psychiatric disorder (particularly mood disorder) is present in 90% of suicides. This explains the risk of suicide of between 47-74% of cases [25, 26, 56]. | Persons with psychiatric disorders account for 14% of school dropouts and 5% of college dropouts [57]. Depression in adolescence is also associated with a higher proportion leaving school without an educational qualification [58]. | Early psychiatric disorder in childhood or adolescence has been investigated across sex groups. Both in Costello 2003 and in Merikangas 2010, boys were more likely to have a psychiatric disorder (Costello: 42.3%; Merikangas: 14.5%) than girls (Costello: 31.0%; Merikangas: 11.6%). Boys and girls differ in the type of disorder: conduct or behavioral disorder are more frequent in boys, whereas depression is more frequent in girls[28, 29]. |
| Impulsiveness | Depressed individuals with high impulsivity scores are more likely to commit suicide [59]; this was significant in 14 out of 20 cohort or case-control studies [60]. | Adolescents with impulsivity or with attention deficit disorder are more likely to face learning problems, not to complete their education, and to be oriented towards a special education track [61]. | A meta-analysis showed that men display a higher risk of impulsivity, a lower punishment sensitivity , a higher reward sensitivity, and a higher sensation-seeking sensitivity than women [30]. |
| Early adversity | There is extensive evidence that childhood adversity is associated with psychotic disorder with a population-attributable risk of 33% [62]. Childhood adversity is also associated with persistence or duration of psychotic disorder, with an OR of 1.8 [63]. A follow-up study of a cohort of half a million Swedes found that childhood adversity was linearly associated with suicide in childhood and early adulthood [46]. | The Swedish cohort study found that childhood adversity was associated with lower school performance [46]. | The proportion of sexual abuse was higher in females than in males, but there were no clear gender differences in any types of abuse or neglect [44, 45]. In the Swedish study, the proportion of females in the group not exposed to any adversity was similar to the proportion in the total sample (48% *vs.* 49%). The effect of emotional mistreatment on mental health and wellbeing was similar for boys and girls [64]. The authors concluded that “no clear (gender) pattern of specific types of symptom outcomes for emotional abuse or emotional neglect was found.” |
| Intelligence | Risk of suicide is lower in those having a higher intelligence test score. [33] | Intelligence is a predictor of educational achievement, with a correlation of 0.69 between the g factor and GCSE score in the UK[65] | The Swedish study was performed with male military conscripts only [33]. No g factor difference between men and women was observed in the UK study, but there was a high level of verbal competence in women than in men. [65] |
