# Supplementary material for: Socioeconomic disparities in suicide: Causation or confounding?
Source: PLoS One. 2021 Jan 4;16(1):e0243895. doi: 10.1371/journal.pone.0243895 (PMC7781379; doi:10.1371/journal.pone.0243895)
Supplement: S2 Table — ¶ Effect of exposure to the reform in the following logit equation: medium high level of education = age group + Census year + Sex + exposure to the reform. (DOCX) [file pone.0243895.s003.docx]

| Country/Region | Year when the law changed | First cohort to be affected | Upper age limit of compulsory education (old to new) | % of person-year exposed to reform (in the census year) | Effect of the reform on the % of those with medium or high level of education  (beta and *P* value)¶ | Source |
| --- | --- | --- | --- | --- | --- | --- |
| Austria | 1948 | 1934 | 14 to 15 | 65.3% (1991) | -0.13 (< .01) | [37] [66] |
| Belgium | 1983 | 1969 | 14 to 18 | 0% (2004) | 0.10 (< .01) | [37] |
| Denmark | 1971 | 1955 | 14 to 16 | 42.9% (2001) | -.20 (< .01) | [67] |
| England-Wales | 1973 | 1957 | 15 to 16 | 40.5% (2006) | .03 (< .01) | [36] |
| Estonia | 1958–1963 | 1945 | 14 to 16 | 53.8% (1998) | 0.07 (< .01) | [67] |
| Finland | 1977 | 1966 | 13 to 16 | 6.2% (2005) | -0.27 (< .01) | [67] |
| Hungary | 1961 | 1947 | 14 to 16 | 54.1% (1999) | 0.09 (< .01) | [68] |
| Italy | 1963 | 1949 | 12 to 15 | 46.8% (2001) | 0.11 (< .01) | [37] |
| Norway | 1960–1972 | 1947-1959 | 14 to 16 | 53.6% (2006) | 0.03 (< .01) | [67] |
| Poland | 1956 |  | 15 to 16 | 59.4% (1991) | 0.09 (< .01) | [69] |
| Spain | 1970 | 1957 | 12 to 14 | 53.2% (2007) | .017 (< .01) | [37] |
| Switzerland | 1940 | 1926 | 14 to 15 | 75.2% (1990) | .02 (< .01)) | [70] |
