# Supplementary material for: Socioeconomic disparities in suicide: Causation or confounding?
Source: PLoS One. 2021 Jan 4;16(1):e0243895. doi: 10.1371/journal.pone.0243895 (PMC7781379; doi:10.1371/journal.pone.0243895)
Supplement: S3 Table — ¶ All estimates are controlled for sex, 5y. age group, and year of the census. Results for all countries are weighted and controlled by country dummies. (DOCX) [file pone.0243895.s004.docx]

|  |  | | | | | |  | |
| --- | --- | --- | --- | --- | --- | --- | --- | --- |
|  | **Risk of suicide in those with a low level of education compared to those with a high level of education, by sex** | | | | | | | |
|  | **Female** | | | **Male** | | |  | |
| **Country** | **RR** | **95%CI** | ***P* value** | **RR** | **95%CI** | ***P* value** | **Khi-2 for Education*Sex** | ***P* value** |
| Austria | 1.21 | (0.80-1.84) | 0.373 | 2.80 | (2.13-3.67) | < .001 | 11.0 | <.001 |
| Belgium | 0.79 | (0.71-0.88) | < .001 | 1.47 | (1.36-1.58) | < .001 | 91.4 | <.001 |
| Denmark | 1.03 | (0.92-1.17) | 0.591 | 1.33 | (1.21-1.46) | < .001 | 10.9 | <.001 |
| England-Wales | 0.99 | (0.48-2.05) | 0.973 | 2.17 | (1.26-3.74) | 0.005 | 2.9 | 0.088 |
| Estonia | 2.25 | (1.71-2.96) | < .001 | 2.96 | (2.49-3.51) | < .001 | 2.8 | 0.094 |
| Finland | 1.26 | (1.13-1.42) | < .001 | 2.16 | (2.00-2.33) | < .001 | 61.0 | <.001 |
| Hungary | 1.96 | (1.74-2.21) | < .001 | 3.96 | (3.66-4.28) | < .001 | 93.2 | <.001 |
| Italy-Turin | 0.68 | (0.42-1.10) | 0.118 | 1.39 | (0.95-2.03) | 0.094 | 5.2 | 0.023 |
| Norway | 0.81 | (0.67-0.98) | 0.028 | 1.77 | (1.54-2.03) | < .001 | 44.3 | <.001 |
| Poland | 2.56 | (2.19-2.99) | < .001 | 4.38 | (4.02-4.76) | < .001 | 35.5 | <.001 |
| Spain (3 Regions) | 1.38 | (1.06-1.81) | 0.017 | 1.97 | (1.66-2.34) | < .001 | 4.9 | 0.027 |
| Switzerland | 0.95 | (0.82-1.10) | 0.512 | 1.71 | (1.57-1.85) | < .001 | 46.6 | <.001 |
| All | 1.32 | (1.26-1.38) | < .001 | 2.51 | (2.44-2.58) | < .001 | 569.8 | <.001 |
